# Effects of dietary butyrate supplementation and crude protein level on carcass traits and meat composition of broiler chickens

**DOI:** 10.5194/aab-62-527-2019

**Published:** 2019-09-02

**Authors:** Gábor Mátis, Janka Petrilla, Anna Kulcsár, Henry van den Bighelaar, Bart Boomsma, Zsuzsanna Neogrády, Hedvig Fébel

**Affiliations:** 1 Division of Biochemistry, Department of Physiology and Biochemistry, University of Veterinary Medicine, István utca 2, 1078 Budapest, Hungary; 2 Palital Feed Additives, De Tweede Geerden 11–13, 5334 LH Velddriel, the Netherlands; 3 Research Institute for Animal Breeding, Nutrition and Meat Science, National Agricultural Research and Innovation Center, Gesztenyés út 1, 2053 Herceghalom, Hungary

## Abstract

The short-chain fatty acid butyrate, either in unprotected or protected form, is widely applied as a growth-promoting feed additive in poultry nutrition; however, its possible effects on the carcass composition of broilers have not been fully elucidated. Further, lowering dietary crude protein (CP) levels is an important issue in poultry farming, contributing to ecologically beneficial lower nitrogen excretion. The main aims of this study were to test how unprotected and protected forms of butyrate and decreased dietary CP content with essential amino acid (lysine, methionine, threonine, tryptophan) supplementation (“LP-EAA” diet) affect carcass parameters and the chemical composition of muscles in broilers. Ross 308 chickens were randomized to seven groups (n=10/group) receiving adequate CP-containing (normal protein, “NP”) or LP-EAA diets, both supplemented with or without unprotected sodium butyrate, and NP diets with different forms of protected sodium butyrate. Carcass traits were measured, and the chemical composition of pectoral and femoral muscles was analyzed at the age of 6 weeks. Carcass weight was significantly increased by the LP-EAA diet and all protected butyrate types tested, while the relative breast meat yield was significantly higher in LP-EAA than NP groups and in both unprotected and protected butyrate-supplemented chickens compared to controls. The protein content of the femoral muscle was significantly decreased, but its lipid content was significantly elevated by the LP-EAA diet and by all types of butyrate addition. However, no changes were detected in the chemical composition of pectoral muscle. In conclusion, breast meat production can be effectively stimulated by dietary factors, such as by reducing dietary CP content with essential amino acid supplementation and by applying butyrate as a feed additive, while its chemical composition remains unchanged, in contrast to the femoral muscle. The aforementioned nutritional strategies seem to be the proper tools to increase carcass yield and to alter meat composition of broilers, contributing to more efficient poultry meat production.

## 1 Introduction

The production of healthy meat is an increasingly important issue worldwide to fulfill the demands of the constantly growing human population. As broiler meat is one of the most common, valuable and easily available protein sources in human nutrition, the significance of efficient poultry husbandry has greatly increased in the last decades. Animal welfare and economic aspects should also be addressed when improving productivity in poultry farming. Nutritional changes, such as altering nutrient composition or applying feed additives may provide great tools for enhancing meat quality and production efficiency. As the application of antibiotics as growth promoters has been prohibited in the EU since 2006 (as described in regulation no. 1831/2003/EC on additives for use in animal nutrition; Phillips, 2007), using alternative feed additives is of particularly high interest in poultry nutrition.

The short-chain fatty acid (n-)butyrate is produced by the microbial fermentation in the ceca. Further, it is widely applied as a growth-promoting feed additive in chicken nutrition. There are several forms of butyrate available, such as unprotected free salts (mostly sodium and calcium butyrate), being absorbed from the crop, the proventriculus and the gizzard (Kulcsár et al., 2017). In contrast, film-coated, fat-embedded or micro-encapsulated protected types provide absorption from the more distal sections of the gastrointestinal tract (Kulcsár et al., 2017). Butyric acid glycerides enable a lipase-driven butyrate release and absorption in the proximal small intestine (Antongiovanni et al., 2007). The main advantage of protected butyrate is that it cannot be absorbed from the proximal gut sections; thus, it can reach the lower small intestine or the hindgut (Kulcsár et al., 2017). Orally applied butyrate can influence small intestinal micromorphology leading to a more efficient nutrient absorption, and positively influence the eubiotic intestinal microflora, increase the gut barrier function and the development of gut-associated lymphoid tissue (Hu and Guo, 2007). Beside these intestinal effects, butyrate as an epigenetically active molecule can cause histone hyperacetylation, described in the liver of chickens after oral butyrate ingestion (Mátis et al., 2013), modulating the expression of certain genes and thus possibly leading to metabolic alterations. For instance, butyrate can affect insulin homeostasis, modifying the abundance of key insulin signaling proteins in a tissue-dependent manner (Mátis et al., 2015; Kulcsár et al., 2016).

Based on its widespread biological actions, butyrate can increase the growth performance of broilers, indicated by a significantly higher body weight gain and decreased feed conversion ratio (FCR) values (Hu and Guo, 2007). It was also reported that butyric acid glycerides increased the carcass weight and breast meat yield of broilers, which effects became even more pronounced under suboptimal health conditions, such as *Eimeria* oocysts (Leeson et al., 2005). However, butyric acid glycerides had no significant effects on the carcass composition of chickens (Antongiovanni et al., 2007). In another study, carcass yield was increased and abdominal fat content was decreased by butyric acid as a feed additive in broilers (Panda et al., 2009). However, it is not known if butyrate as a feed additive, either as an unprotected free salt or in a protected form, may influence muscle composition and thus the meat quality of broilers.

Altering dietary crude protein (CP) content and free amino acid supplementation can also affect growth and meat production of broilers. Decreased dietary CP levels could cause impaired body weight gain and FCR if the digestible amino acid profile is unbalanced or an amino acid is limiting (Aftab et al., 2006). However, this effect could be ameliorated by the supplementation of the diet with limiting free amino acids, maintaining physiological productivity (Aletor et al., 2000). The application of low-CP diets increased the fat content of the carcass (Bregendahl et al., 2002) but decreased the nitrogen excretion, the latter being highly beneficial for the environment (Aletor et al., 2000). Similarly, the abdominal fat mass and the lipid content of the carcass were both significantly increased in chickens fed a low-CP diet. This effect was overcome when arginine and lysine were supplemented, indicating that arginine and lysine concentrations were deficient before the supplementation (Hurwitz et al., 1998). The increasing action of low-CP diets on the fat mass of the carcass is also mainly associated with the unbalanced dietary amino acid profile because excess amino acids are broken down and may be utilized for triacylglycerol synthesis (Wu et al., 2014). However, abdominal fat mass was reduced when low-CP and low metabolizable energy (ME) diets were fed to broiler chickens (Cornejo et al., 1991). Lowering dietary CP and ME content also decreased the final body weight but increased the relative weight of thighs and wings in broilers (Delezie et al., 2010).

The main goal of this study was to assess if unprotected sodium butyrate or various protected (film-coated and fat-embedded) sodium butyrate types may influence carcass characteristics and muscle composition of broiler chickens. Further, the suggested effects of reduced dietary CP levels with essential amino acid supplementation were also to be analyzed to optimize the dietary conditions required for sustainable and economic broiler meat production. By combining these two factors, we wanted to monitor if unprotected sodium butyrate as a pure substance has different effects on carcass traits under different dietary conditions, such as normal or lowered CP levels, possibly due to the suggested interplay of dietary amino acid supply, composition of the gut flora, and microbial butyrate utilization or production.

## 2 Materials and methods

### 2.1 Ethics statement

The housing and treatment of the animals were carried out in accordance with the national and international laws as well as with the institutional guidelines. Experimental procedures were approved by the Government Office of Pest County, Food Chain Safety, Plant Protection and Soil Conservation Directorate, Budapest, Hungary (number of permission: PEI/001/1430-4/2015).

**Table 1:** Experimental groups of the trial.

Experimental group	Diet type	Butyrate supplementation
NP CTR	normal dietary crude protein level	–
NP SB	normal dietary crude protein level	unprotected sodium butyrate (1.5 g kg${}^{-1}$ diet)
NP S90	normal dietary crude protein level	film-coated sodium butyrate, Intest-Plus S90 (1.0 g kg${}^{-1}$ diet)
NP SC40	normal dietary crude protein level	vegetable fat-embedded sodium butyrate, Intest-Plus SC40 (1.5 g kg${}^{-1}$ diet)
NP SC30	normal dietary crude protein level	vegetable fat-embedded sodium butyrate, Intest-Plus SC30 (2.0 g kg${}^{-1}$ diet)
LP-EAA CTR	low-protein diet supplemented with essential amino acid (L-lysine, DL-methionine, L-threonine and L-tryptophan)	–
LP-EAA SB	low-protein diet supplemented with essential amino acid (L-lysine, DL-methionine, L-threonine and L-tryptophan)	unprotected sodium butyrate (1.5 g kg${}^{-1}$ diet)

### 2.2 Animals and treatments

Day-of-hatch male Ross 308 broiler chicks, obtained from a commercial hatchery (Gallus Company, Devecser, Hungary), were randomly allocated to seven dietary groups (n=10 per group, Table 1). The animals were housed in metal pens on wheat straw litter in the Research Institute for Animal Breeding, Nutrition and Meat Science, National Agricultural Research and Innovation Center (Herceghalom, Hungary). Environmental conditions were set according to the recommendations of the breeder (Aviagen, 2014); feed and drinking water were provided ad libitum during the entire study.

Five groups were fed with diets containing the normal dietary CP level of the appropriate dietary phase (Table 1, “normal protein”; NP groups with 22.7 %, 21.4 % and 19.1 % CP in starter, grower and finisher diets, respectively). Two groups of chickens received a low-CP, amino-acid-supplemented (L-lysine, DL-methionine, L-threonine and L-tryptophan) diet (Table 1, “low protein, essential amino acid supplemented”; LP-EAA groups with 19.1 %, 18.0 % and 16.0 % CP in starter, grower and finisher phase, respectively). The amino acid levels in all diets were calculated, and the four first-limiting commercially available amino acids (Lys, Met, Thr, Trp) were supplemented to meet the recommendations of the breeder (Aviagen, 2014). Starter diets were switched to growers on day 10, while growers were switched to finishers on day 25. All diets within a phase were set to be isoenergetic. Ingredients and calculated and measured nutrient composition of diets are shown in Table 2.

**Table 2:** Ingredients and nutrient composition of diets.

		Starter		Grower		Finisher	
Ingredients		NP	LP-EAA	NP	LP-EAA	NP	LP-EAA
Maize	%	57.60	61.00	60.71	65.31	63.66	70.25
Extr. soybean meal	%	27.00	28.00	22.20	24.54	24.50	20.29
PL-68${}^{1}$	%	6.50	0	8.00	1.00	3.00	0.70
Sunflower oil	%	3.50	3.50	4.80	4.50	5.00	4.30
Wheat bran	%	0	1.72	0	0	0	0
Limestone	%	1.70	1.60	1.30	1.20	1.09	1.09
Monocalcium phosphate	%	1.80	2.00	1.35	1.60	1.40	1.60
Salt	%	0.40	0.40	0.40	0.40	0.40	0.40
L-lysine	%	0.44	0.58	0.34	0.41	0.19	0.39
DL-methionine	%	0.43	0.44	0.36	0.37	0.26	0.33
L-threonine	%	0.09	0.22	0	0.15	0	0.13
L-tryptophan	%	0.04	0.04	0.04	0.02	0	0.02
Vitamin and mineral premix${}^{2}$	%	0.50	0.50	0.50	0.50	0.50	0.50
Calculated values (as-fed basis)							
AME${}_{n}$	MJ kg${}^{-1}$	12.64	12.61	13.08	13.04	13.12	13.12
Lysine	%	1.44	1.48	1.27	1.24	1.11	1.12
Methionine + cysteine	%	1.07	1.05	0.96	0.95	0.86	0.87
Threonine	%	0.97	0.94	0.84	0.84	0.74	0.74
Tryptophan	%	0.23	0.25	0.21	0.20	0.19	0.18
Arginine	%	1.28	1.24	1.19	1.11	1.11	0.99
Isoleucine	%	0.87	0.78	0.82	0.72	0.75	0.64
Leucine	%	1.59	1.53	1.52	1.48	1.50	1.39
Valine	%	1.01	0.88	0.97	0.81	0.87	0.74
Total Ca	%	1.15	1.15	0.92	0.93	0.85	0.87
Total P	%	0.79	0.80	0.68	0.69	0.66	0.68
Available P	%	0.54	0.53	0.45	0.45	0.42	0.44
Analyzed values (as-fed basis)							
Dry matter	%	90.30	90.79	90.40	90.69	91.02	90.80
Crude protein	%	22.69	19.07	21.36	18.01	19.08	16.03
Ether extract	%	6.78	6.53	7.42	7.36	7.62	7.57
Crude fiber	%	2.43	2.70	2.20	2.41	2.56	2.45
Ash	%	6.45	6.56	5.45	5.52	5.28	5.30
Starch	%	38.24	41.75	41.00	43.70	42.80	45.93
Sugar	%	3.17	3.47	2.86	3.13	3.12	2.85

Abbreviations used: NP – normal protein diet with normal dietary crude protein levels; LP-EAA – low-protein diet supplemented with essential amino acid (L-lysine, DL-methionine, L-threonine and L-tryptophan); AME${}_{n}$ – nitrogen-corrected apparent metabolizable energy; AME${}_{n}$ (per kg) =0.155×% crude protein + 0.343×% ether extract + 0.1669×% starch + 0.130×% sugar (Commission Regulation (EC) no. 152/2009). ${}^{1}$ Protein concentrate, by-product of glutamic acid production from bacterial biomass (KJK-Agroteam Ltd., Hungary). Amino acid contents: L-lysine 2.4 %, DL-methionine 0.9 %, L-cysteine 0.2 %, L-threonine 3.0 %, L-proline: 1.6 %, L-arginine 3.1 %, L-serine 2.4 %, L-glutamine 12.3 %, L-tryptophan 0.69 %, glycine 2.4 %, L-alanine 6.0 %, L-valine 3.2 %, L-isoleucine 2.4%, L-leucine 3.1 %, L-phenylalanine 2.2 %, L-histidine 0.9 %, L-aspartic acid 5.5 %. ${}^{2}$ Per kilogram of diet: vitamin A 12 013 IU; vitamin D${}_{3}$ 3875 IU; vitamin K 3.3 mg; vitamin E 46.5 IU; vitamin B1 2.33 mg; vitamin B2 7.44 mg; vitamin B6 3.88 mg, vitamin B12 0.016 mg; calcium pantothenate 13.95 mg; folic acid 1.56 mg; niacin 46.5 mg; choline chloride 504 mg; Fe 60 mg; Mn 100 mg; Cu 12.5 mg; Zn 83 mg; Se 0.42 mg; Co 0.28 mg; I 1.25 mg.

**Table 3:** Body weight values of chickens at different time points.

		Abbreviation of dietary group							
Parameter	Week	NP CTR	NP SB	NP S90	NP SC40	NP SC30	LP-EAA CTR	LP-EAA SB	Significant differences
Body weight (g)	1	171.0	179.8	184.3	180.6	177.9	174.9	179.6	
		±8.4	±4.9	±3.3	±5.2	±4.5	±7.1	±8.1	
	3	679.5	638.1	800.0	794.3	821.5	824.3	864.0	${}^{***}$ LP-EAA vs. NP, ${}^{***}$ NP S90, NP
		±20.8	±34.1	±24.7	±22.1	±21.3	±32.9	±28.6	SC40, NP SC30 vs. NP CTR
	6	2234.5	2315.6	2738	2719	2700	2934.7	2635.3	${}^{***}$ LP-EAA vs. NP, ${}^{***}$ NP S90, NP
		±97.5	±117.8	±84.3	±103.5	±66.4	±47.9	±97.0	SC40, NP SC30 vs. NP CTR

The abbreviations of the experimental groups are indicated in Table 1. Results are expressed as mean ± SE. Significant differences revealed as main effects (LP-EAA vs. NP) or by post hoc tests (NP S90, NP SC40, NP SC30 vs. NP CTR) are marked in the following way: ${}^{***}P<0.001$.

The feed of two groups (one within the NP and one within the LP-EAA dietary groups) was supplemented with unprotected sodium butyrate (1.5 g kg${}^{-1}$ diet, dosage set as the average concentration used in poultry nutrition; Sigma-Aldrich, Munich, Germany), indicated as NP SB and LP-EAA SB. Further, different forms of protected sodium butyrate were added to three NP groups as follows: a highly concentrated, film-coated sodium butyrate: Intest-Plus S90 with 90 % sodium butyrate content, 1.0 g kg${}^{-1}$ diet (pure sodium butyrate content: 0.9 g kg${}^{-1}$ diet), indicated as NP S90 group; vegetable fat-embedded sodium butyrate products with various butyrate contents: Intest-Plus SC40 with 40 % sodium butyrate content, 1.5 g kg${}^{-1}$ diet (pure sodium butyrate content: 0.6 g kg${}^{-1}$ diet), indicated as NP SC40 group; Intest-Plus SC30 with 30 % sodium butyrate content, 2.0 g kg${}^{-1}$ diet (pure sodium butyrate content: 0.6 g kg${}^{-1}$ diet), abbreviated as NP SC30 group. Protected butyrate products were obtained from Palital Feed Additives (Velddriel, the Netherlands); doses were set according to the manufacturer's instructions. Groups without dietary butyrate supplementation were regarded as controls (NP CTR group with normal dietary CP levels, LP-EAA CTR group kept on a low-CP, amino-acid-supplemented diet). An overview of the experimental groups is presented in Table 1.

### 2.3 Samplings, measurements and chemical analyses

Chickens were slaughtered on day 42 by decapitation after applying carbon dioxide. Thereafter, the following parameters of carcass traits were measured: carcass weight (including skin and wings, excluding giblets), deboned breast meat yield, femoral muscle weight, liver weight, heart weight, spleen weight and abdominal fat weight. Deboned breast meat yield, femoral muscle weight and abdominal fat weight were additionally evaluated relative to body weight to obtain information of the proportion of carcass parts.

Representative samples of pectoral and femoral muscle (60 g tissue was taken always from the same anatomic site) were taken for chemical analysis of meat composition. Muscle samples were minced, freeze-dried, ground and stored at -20 ${}^{\circ }$C until further processing. Chemical analyses were conducted as outlined by the Association of Analytical Communities (AOAC, 1990). After thawing, dry matter content was measured by drying the samples at 135 ${}^{\circ }$C for 2 h according to the appropriate AOAC protocol (method number 930.15). Crude protein content of muscle samples was analyzed by the Kjeldahl procedure (AOAC method number 920.39); lipid content was determined as ether extract using a Soxhlet apparatus (AOAC method number 988.05).

Diet samples were ground in a hammer mill with a 1 mm screen and analyzed in duplicate for dry matter (dm), ash, crude protein (N×6.25), crude fiber, ether extract and starch according to the methods of AOAC (1990).

### 2.4 Statistical analyses

Data were statistically analyzed with the R 3.2.2 software, by using multi-way ANOVA to consider the effects of independent variables (dietary CP levels and different butyrate supplementations) on the measured parameters as response (dependent) variables. A post hoc Tukey's test was applied for pairwise comparisons. Data were normally distributed and within-group variances were homogenous. Differences between groups were regarded as statistically significant when P<0.05. In the Results section, P values gained by post hoc tests are presented. Results are expressed as mean ± standard error (SE).

## 3 Results

The body weight and feed intake of the animals matched the standards of the Ross technology in all phases of broiler fattening. Data on body weights are presented in Table 3, showing significantly higher body weight values in LP-EAA birds than in NP groups at weeks 3 and 6 (P<0.001 in both cases). The body weight of the chickens was significantly higher in groups receiving protected butyrate supplementation (NP S90, NP SC40 and NP SC30 groups) compared to controls (NP CTR group) at week 3 and 6 (P<0.001 at both time points).

**Figure 1:**
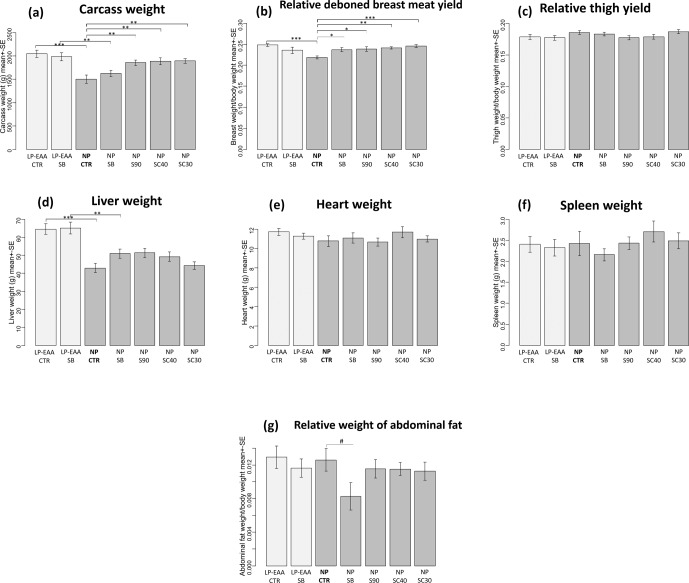
Results of carcass trait measurements. **(a)** Carcass weight. **(b)** Relative deboned breast meat yield. **(c)** Relative thigh yield. **(d)** Liver weight. **(e)** Heart weight. **(f)** Spleen weight. **(g)** Relative weight of abdominal fat. The abbreviations of the experimental groups are indicated in Table 1. Results are expressed as mean ± SE. Significant differences revealed by post hoc tests are marked with the following symbols: #P<0.10; ${}^{*}\;P<0.05$; ${}^{**}\;P<0.01$; ${}^{***}\;P<0.001$.

Carcass weight (Fig. 1a) was significantly increased by the low-protein diet with essential amino acid supplementation: significantly higher values were measured in the LP-EAA CTR group than in NP CTR animals (P<0.001) and in the LP-EAA SB than in the NP SB group (P=0.005). No significant difference was observed between unprotected sodium-butyrate-supplemented and control groups. All protected sodium butyrate products (fed in NP S90, NP SC40 and NP SC30 groups) significantly increased carcass weight compared to controls (NP CTR group) (P=0.009, P=0.003 and P=0.002, respectively).

The relative weight of deboned breast meat (Fig. 1b) was significantly greater in the LP-EAA CTR than in the NP CTR group (P<0.001). All forms of butyrate significantly (NP SB: P=0.046; NP S90: P=0.003; NP SC40: P=0.007; and NP SC30: P<0.001) elevated breast meat yield when compared to controls (NP CTR group). In contrast to the breast meat, no significant differences were observed regarding the relative mass of thighs (Fig. 1c) between any experimental groups.

Liver weight (Fig. 1d) was significantly greater in LP-EAA CTR than in NP CTR animals (P<0.001) and in LP-EAA SB than in NP SB chickens (P=0.003). In the case of further giblets (heart and spleen, Fig. 1e–f, respectively), no significant differences were detected. Relative abdominal fat mass (Fig. 1g) tended to be decreased by unprotected sodium butyrate (NP SB compared to the NP CTR group: P=0.077), but no significant effects were found with regard to dietary CP levels or protected sodium butyrate products.

**Figure 2:**
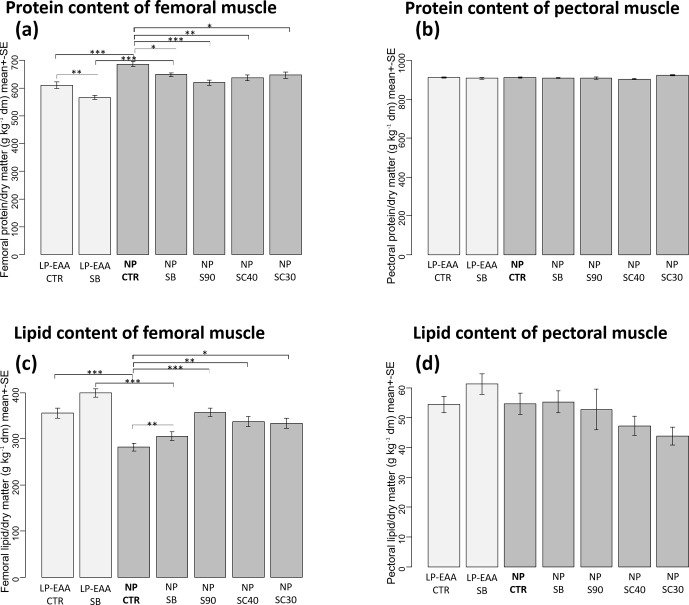
Results of chemical analysis of muscle composition. **(a)** Protein content of the femoral muscle. **(b)** Protein content of pectoral muscle. **(c)** Lipid content of the femoral muscle. **(d)** Lipid content of pectoral muscle. The abbreviations of the experimental groups are indicated in Table 1. Results are expressed as mean ± SE in grams per kilograms of dry matter (dm). Significant differences revealed by post hoc tests are marked in the following way: ${}^{*}\;P<0.05$; ${}^{**}\;P<0.01$; ${}^{***}\;P<0.001$.

The chemical analysis of muscle composition revealed that the protein content of the femoral muscle (Fig. 2a) was significantly decreased by the essential-amino-acid-supplemented low-CP diet and by all forms of butyrate used. In detail, a significantly decreased protein content was measured in the LP-EAA CTR compared to the NP CTR groups (P<0.001) and, similarly, in the thighs of LP-EAA SB chicks compared to those of NP SB animals (P<0.001). The significant lowering effect of unprotected sodium butyrate could be observed between NP SB and NP CTR groups (P=0.031) and between LP-EAA SB and LP-EAA CTR groups (P=0.008) as well. A significant reduction in femoral protein content was also measured in the case of all types of protected sodium butyrate applied (NP S90: P<0.001; NP SC40: P=0.002; and NP SC30: P=0.02) when compared to control animals (NP CTR group). The protein content of pectoral muscle (Fig. 2b) remained unchanged in all experimental groups.

The lipid content of the femoral muscle (Fig. 2c) was significantly affected by the dietary CP level and by butyrate supplementation as well. Significantly higher values were measured in the thighs of chickens kept on a low-CP, amino-acid-supplemented diet than in those of the normal CP groups (LP-EAA CTR compared to NP CTR group: P<0.001; LP-EAA SB compared to NP SB animals: P<0.001). All types of sodium butyrate supplementation significantly elevated the lipid content of the femoral muscle (NP SB: P=0.018; NP S90: P<0.001; NP SC40: P=0.001; and NP SC30: P=0.003) when compared to butyrate-free controls (NP CTR group). No significant changes were detected in the lipid content of breast meat (Fig. 2d) between any trial groups.

## 4 Discussion

Butyrate as a feed additive, both in unprotected or protected form, had a stimulatory action on the growth of broilers and had a remarkable influence on the chemical composition of thighs. Our results showed that live weight and carcass weight were significantly increased by all protected butyrate forms, and relative breast meat yield was elevated in chickens fed with unprotected and protected butyrate as well, compared to controls without butyrate addition.

The stimulatory action of butyrate on broiler meat production has been already described in several studies, showing increased carcass yield of butyrate-supplemented animals (Leeson et al., 2005; Hu and Guo, 2007; Panda et al., 2009). In our study, the applied protected butyrate products were able to increase carcass weight, being more potent to increase meat production than unprotected butyrate, while the latter could not provoke a significant elevation in carcass yield. Relative breast meat yield was increased by both unprotected and protected butyrate supplementation, indicating higher mass and a higher proportion of pectoral muscle among meat types. The absolute breast meat mass, as a sum of the increased carcass weight and relative breast meat yield, was also elevated (with approximately 34 %) by all types of protected butyrate application (breast meat mass in butyrate-free controls, NP CTR group: 491.4±33.0 g; in NP S90 group: 655.3±29.9; in NP SC40 group: 657.0±29.1 g; in NP SC30 group: 663.6±19.3 g).

The growth-promoting action of butyrate can be related to its several biological effects. In the intestines, butyrate stimulates the development of the intestinal mucosa, increases the barrier function of the gut wall and maintains intestinal microflora by selectively inhibiting the growth of certain pathogenic bacteria (Hu and Guo, 2007). The greater absorptive capacity and more balanced microflora may contribute to increased growth and altered carcass characteristics. In addition, the absorbed butyrate can act in several tissues as an epigenetically active molecule and may also elicit some receptor-mediated effects (Mátis et al., 2013). For instance, the butyrate-associated modulation of insulin homeostasis can also be related to stimulated muscle development as insulin receptor β was selectively upregulated in skeletal muscle after oral butyrate application, resulting in increased insulin sensitivity (Mátis et al., 2015).

The lipid content of the femoral muscle was increased and the protein content was decreased by all types of butyrate applied, but no changes were observed in the chemical composition of breast meat. The observed alterations in thighs may improve meat quality, and the increased muscular lipogenesis was not coupled with abdominal fat deposition. A similar action of orally applied butyrate was also described in feedlot cattle, where the marbling of the meat was significantly increased by calcium butyrate as a feed additive (Moreira et al., 2016).

No relevant differences were found between the efficacy of different protected butyrate types; the tested protected products seemed to be more effective in some cases than unprotected free butyrate, such as in stimulating carcass weight (which effect was lacking in the case of unprotected butyrate) or in increasing breast meat yield to a higher extent than unprotected butyrate. Dietary supplementation of protected butyrate provides butyrate release in more distal sections of the intestines, while unprotected butyrate is rapidly absorbed from the proximal section of the gastrointestinal tract (Kulcsár et al., 2017). These different kinetic properties of protected butyrate products should deliver better butyrate exposure for the intestinal microflora, and the prolonged absorption may also have differing effects on various extraintestinal tissues compared to the action of unprotected butyrate (Kulcsár et al., 2016, 2017; Petrilla et al., 2018).

According to our results, the low-CP, amino-acid-supplemented diet increased the live body weight (at week 3 and 6), carcass weight and breast meat yield of broilers. In general, reduced dietary CP levels with improper amino acid composition can diminish the growth of chickens due to the inadequate amino acid supply (Aletor et al., 2000). However, no decrease can be found in growth performance when providing amino acids in a well-balanced profile, possibly ensured by essential amino acid supplementation (Aletor et al., 2000). Similarly to our results, significantly greater body weights were measured in chickens kept on a low-protein diet, supplemented with limiting amino acids by Khan et al. (2011). This effect is suggested to be connected to more free essential amino acids being available for the amino-acid-supplemented groups compared to the animals fed with normal protein diets. It was also reported by Awad et al. (2014) that dietary CP levels could be lowered to a limited extent together with essential amino acid supplementation to maintain the normal growth and health of broilers. The supplementation of free amino acids to broiler feeds is a key factor in lowering dietary CP levels (Pesti, 2009), reducing nitrogen excretion but maintaining or even increasing growth and meat production. The elevation of the relative breast meat yield in broilers kept on an LP-EAA diet indicated that not only the absolute mass but also the proportion of breast meat as the most valuable part of the chicken carcass was increased by the low-CP and amino-acid-supplemented diet.

In our study, the weight of the liver was significantly increased by the essential amino-acid-supplemented low-CP diet. In contrast, Awad et al. (2014) found no changes in liver mass when low-protein and amino-acid-supplemented diets were given to broilers; however, they investigated chickens in the grower phase and applied slightly lower dietary lysine levels compared to our study. As observed in the present study, the protein content of the femoral muscle was significantly decreased, while its lipid content was increased by the LP-EAA diet when compared to NP animals. However, relative abdominal fat mass was not affected by dietary CP levels. Presumably due to the lower dietary protein and higher carbohydrate content (to gain isoenergetic diets), the lipogenesis could be stimulated in low-CP groups. However, the subsequently increased triacylglycerol disposition was realized only in femoral muscle improving meat quality and did not result in greater abdominal fat reserves. In another study, various isoenergetic low-CP diets (from 23 % to 18%) increased the lipid content of the whole carcass independently of the provided amino acid supplementation in 3-week-old broilers (Bregendahl et al., 2002). Similarly, abdominal fat mass and the amount of extractable carcass fat were increased by lower dietary CP levels (from 25 % to 18 %) but were reduced by arginine or lysine supplementation in the starter phase (Hurwitz et al., 1998).

The effects of unprotected sodium butyrate as a pure substance were compared in chickens kept on an NP or LP-EAA diet to gain some preliminary data about the possible interaction of various dietary factors. According to our results it can be observed that butyrate similarly altered the chemical composition of the femoral muscle in both cases. However, the stimulatory action of unprotected butyrate on breast meat yield was lacking in the case of the lowered CP supply with essential amino acid supplementation. The partly different action of butyrate in normal and low-CP diets cannot be explained based on these data. However, it can be hypothesized that a different amino acid supply may alter the composition of the intestinal microflora, possibly interfering with the utilization of exogenously applied butyrate and the endogenous microbial butyrate production. Further, orally applied butyrate may also influence the pH of the ingesta, possibly acting on protein digestion and utilization as well. Based on our results, it can be highlighted that feed additives such as butyrate can elicit different effects under various dietary conditions; thus, combining more nutrition strategies to optimize animal production should be considered carefully. Therefore, based on these initial results about the combination of pure sodium butyrate and an altered dietary CP supply, future studies are needed with regard to the possible interaction of different butyrate-containing products and dietary CP levels or cereal types.

It has to be stressed that, in contrast to the thighs, no diet-associated changes (either in the case of butyrate addition or dietary CP levels) could be detected in the chemical composition of the pectoral muscle. This finding might be connected to different muscle fiber composition and metabolic properties of various muscles and suggests a great stability of breast meat composition.

Based on our results, it can be concluded that the development and production of breast meat can be effectively stimulated by the dietary CP content and butyrate supplementation, but its chemical composition remains unchanged at the same time. The application of unprotected or protected butyrate as feed additives and decreased dietary CP levels with concomitant essential amino acid supplementation seem to be nutritional tools to increase carcass yield and to alter the meat composition of broilers.

## Data Availability

The original data of the paper are available upon request from the corresponding author.
